# Exploring optimal examination to detect occult anastomotic leakage after rectal resection in patients with diverting stoma

**DOI:** 10.1186/s12893-020-00706-x

**Published:** 2020-03-19

**Authors:** Daichi Kitaguchi, Tsuyoshi Enomoto, Yusuke Ohara, Yohei Owada, Katsuji Hisakura, Yoshimasa Akashi, Kazuhiro Takahashi, Koichi Ogawa, Osamu Shimomura, Tatsuya Oda

**Affiliations:** Department of Gastrointestinal and Hepato-Biliary-Pancreatic Surgery, Faculty of Medicine, University of Tsukuba, 1-1-1, Tennodai, Tsukuba, Ibaraki, 305-8575 Japan

**Keywords:** Diverting stoma, Loop ileostomy, Anastomotic leakage, Rectal resection, Early closure

## Abstract

**Background:**

When considering “early stoma closure”, both standardized inclusion/exclusion criteria and standardized methods to assess anastomosis are necessary to reduce the risk of occult anastomotic leakage (AL). However, in the immediate postoperative period, neither have the incidence and risk factors of occult AL in patients with diverting stoma (DS) been clarified nor have methods to assess anastomosis been standardized. The aim of this study was to elucidate the incidence and risk factors of occult AL in patients who had undergone rectal resection with DS and to evaluate the significance of computed tomography (CT) following water-soluble contrast enema (CE) to detect occult anastomotic leakage.

**Methods:**

This was a single institutional prospective observational study of patients who had undergone rectal resection with the selective use of DS between May and October 2019. Fifteen patients had undergone CE and CT to assess for AL on postoperative day (POD) 7, and CT was performed just after CE. Univariate analysis was performed to assess the relationship between preoperative variables and the incidence of occult AL on POD 7.

**Results:**

The incidence of occult AL on postoperative day 7 was 6 of 15 (40%). Hand-sewn anastomosis, compared with stapled anastomosis, was a significant risk factor. Five more cases with occult AL that could not be detected with CE could be detected on CT following CE; CE alone had a 33% false-negative radiological result rate.

**Conclusions:**

Hand-sewn anastomosis appeared to be a risk factor for occult AL, and CE alone had a high false-negative radiological result rate. When considering the introduction of early stoma closure, stapled anastomosis and CT following CE could be an appropriate inclusion criterion and preoperative examination, respectively.

## Background

Diverting stoma (DS) is used primarily to protect the anastomosis and prevent pelvic sepsis after rectal surgery [1]. Several studies have shown that a particular benefit of DS is the reduction in the number of leaks requiring surgery [2–6]. As a DS, loop ileostomy is preferred to colostomy by most surgeons because the former is easy to construct and close without the risk of injury to the colic vascular arcade, and there are fewer problems with prolapse [7].

Previous studies have shown complications of DS with rates up to 43% related to the loop ileostomy, including outlet obstruction, readmissions, dehydration due to high output, and chronic renal failure [8–10]. Although the majority of them have been classified as Clavien-Dindo Grade I, some complications can be severe. In addition, patients with rectal cancer are increasingly being offered postoperative adjuvant therapy, which creates uncertainty about the timing of DS closure [11].

A multicenter randomized controlled trial, EASY, showed that early DS closure (EC) significantly reduced postoperative morbidity, especially stoma-related complications, including skin irritation, stomal ulcer, and leakage outside the appliance bag [12]. A meta-analysis also showed that there was no significant difference between EC and late stoma closure group in the incidence of anastomotic leakage (AL) and reoperation [13]. However, in the literature, approximately one-third of patients were deemed inappropriate for early reversal [14, 15]; in the EASY trial, the exclusion rates reached two-thirds of patients because of the strict exclusion criteria, including diabetes mellitus (DM) and steroid treatment [12].

EC has not been widely adopted as a standard treatment strategy. In the early postoperative period, the incidence and risk factors of occult AL in patients who had undergone rectal resection with DS have not been clarified. Methods to assess anastomosis have also not been standardized, and a false-negative radiological result may lead to performing potentially dangerous EC for patients with occult AL, thereby increasing the risk of anastomotic septic complications. Therefore, both standardized inclusion and exclusion criteria and standardized methods to assess anastomosis are necessary to perform EC safely.

The aim of this study was to clarify the incidence and risk factors of occult AL in patients who had undergone rectal resection with DS and to consider the significance of computed tomography following water-soluble contrast enema to detect occult AL.

## Methods

### Demographics

All patients who had undergone rectal resection with DS between May and October 2019 were enrolled in our prospective, single-center institutional database. The standard indication for DS at our institute is that the anastomotic level from the anal verge is approximately 5 cm or less. However, when special events occur (e.g., intraoperative colorectal injury) and there is strong concern for anastomotic leakage, DS is sometimes constructed. All patients were scheduled to undergo stoma closure several months after the initial surgery, and the subsequent treatment strategy was not affected by the results of this study.

All patients underwent digital rectal examination (DRE), water-soluble contrast enema (CE), and computed tomography (CT) to assess the anastomosis. These three examinations were performed on postoperative day (POD) 7, and CT was performed just after CE. Hematological examinations, including estimation of white blood cell counts (WBC) and C-reactive protein (CRP) levels, were also performed on POD 7 to assess inflammatory status.

CE was performed by the same experienced colorectal surgeon using 100% Gastrografin® as the contrast medium. The contrast medium was instilled through a catheter placed in the rectum just below the anastomosis. The amount of contrast medium infused was 50–100 mL, and when leakage of contrast medium was detected extraluminally, the examination was immediately stopped.

**Fig. 1:**
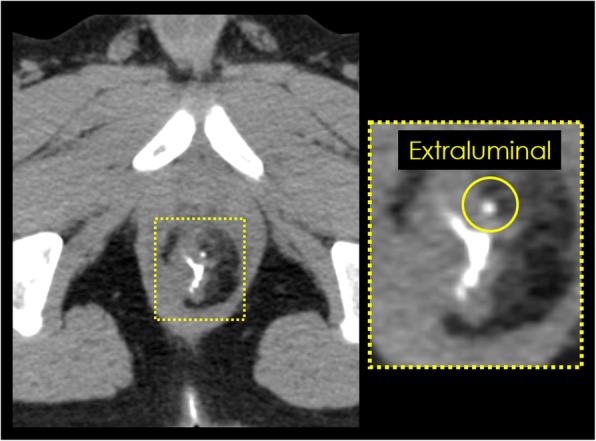
Pelvic plain computed tomography (CT) without contrast medium. Retrograde infusion of Gastrografin® was detected extraluminally on CT image, and this case was diagnosed as an occult anastomotic leakage

Pelvic plain CT without contrast medium was performed, and when retrograde infusion of Gastrografin® was detected extraluminally on CT image, the case was diagnosed as an occult anastomotic leakage (Fig. 1).

Data were collected through electronic medical record systems. Patient characteristics included age, sex, body mass index (BMI), previous abdominal operations, American Society of Anesthesiology physical status (ASA-PS) classification, smoking history, DM, and primary disease. Treatment characteristics included preoperative treatment, operation, minimally invasive surgery (MIS), anastomosis, and distance of the anastomosis from the anal verge (AV).

### Statistical analysis

Quantitative data were reported as median [range] and compared using the Mann-Whitney U test. Qualitative data were reported as the number of patients (percentage) and compared using Fisher’s exact test, as appropriate. All tests were two-sided, with the level of significance set at *p* < 0.05. All statistical analyses were performed with EZR (Saitama Medical Center, Jichi Medical University, Saitama, Japan), which is a graphical user interface for R (The R Foundation for Statistical Computing, Vienna, Austria). EZR is a modified version of R commander designed for statistical functions frequently used in biostatistics [16]. We used statistics in a descriptive fashion realizing that with the number of subjects, no robust statistical analyses were possible.

## Results

During the study period, 15 patients underwent rectal resection with a DS. Patient characteristics are detailed in Table 1.

**Table 1:** Patient characteristics

	*n* = 15
Age (years)	56 [27–70]
Sex (male)	12 (80%)
BMI (kg/m^2^)	24 [18–39]
ASA-PS	
1	2 (13%)
2	10 (67%)
3	3 (20%)
Smoking	10 (67%)
DM	2 (13%)
Primary disease	
Rectal cancer	10 (67%)
UC	4 (27%)
FAP	1 (7%)

*BMI* body mass index; *ASA-PS* American Society of Anesthesiology physical status; *DM* diabetes mellitus; *UC* ulcerative colitis; *FAP* familial adenomatous polyposis

The median age of the cohort was 56 years [27–70], with 80% of patients being male. The median BMI was 24 kg/m^2^ [18–39], and most (67%) patients’ ASA-PS classification was 2.

Ten patients had a history of smoking, and 2 patients had DM. Regarding primary disease, 10 patients had rectal cancer, 4 patients had ulcerative colitis (UC), and one patient had familial adenomatous polyposis (FAP).

Treatment characteristics are detailed in Table 2. Three patients underwent chemoradiotherapy (CRT), and 2 patients were treated with a steroid. Regarding the type of operation, low anterior resection (LAR), intersphincteric resection (ISR), and total colectomy with ileal pouch-anal anastomosis (IPAA) were performed for 4 patients, 6 patients, and 5 patients, respectively. A transanal total mesorectal excision (TaTME) was performed for 8 patients, and robotic surgery was performed in only one case.

**Table 2:** Treatment characteristics

	n = 15
Preoperative treatment	
NAC	1 (7%)
CRT	3 (20%)
Steroid	2 (13%)
Operation	
LAR	4 (27%)
ISR	6 (40%)
IPAA	5 (33%)
MIS	
Laparoscopy	12 (80%)
TaTME	8 (53%)
Robot	1 (7%)
Anastomosis	
Stapled	8 (53%)
Hand-sewn	7 (47%)
Distance of anastomosis (cm from AV)	4 [2–6]

*NAC* neoadjuvant chemotherapy; *CRT* chemoradiotherapy; *LAR* low anterior resection; *ISR* intersphincteric resection; *IPAA* total colectomy with ileal pouch-anal anastomosis; *MIS* minimally invasive surgery; *TaTME* transanal total mesorectal excision; *AV* anal verge

The number of patients with stapled anastomosis and hand-sewn anastomosis were 8 and 7, respectively, and the median distance of anastomosis from the AV was 4 cm [2–6].

### Incidence and risk factors of occult AL

With DRE alone, it was difficult to detect occult AL. Even with water-soluble CE, only one case (7%) of occult AL was successfully detected in this study. With CT following CE, the incidence of occult AL on POD 7 increased to 40%.

The relationship between each variable and the incidence of occult AL on POD 7 is summarized in Table 3. The incidence of occult AL was 71% in the hand-sewn anastomosis group and 13% in the stapled anastomosis group, which reached a statistically significant difference (*p = 0.0406*). The other variables had no statistically significant correlation with the incidence of occult AL.

**Table 3:** Relationship between each variable and the incidence of occult AL on POD 7

		n	Occult AL	OR [95%CI]	P value*
Age (years)	< 60	9	5 (56%)	0.181	*0.287*
	60≤	6	1 (17%)	[0.003–2.69]	
Sex	Male	12	5 (42%)	1.40	*1*
	Female	3	1 (33%)	[0.057–101]	
BMI (kg/m^2^)	< 25	8	3 (38%)	1.23	*1*
	25≤	7	3 (43%)	[0.101–15.4]	
ASA-PS	1–2	12	4 (33%)	3.62	*0.525*
	3	3	2 (67%)	[0.147–264]	
Smoking	Yes	10	4 (40%)	1	*1*
	No	5	2 (40%)	[0.073–17.3]	
DM	Yes	2	0	0	*0.486*
	No	13	6 (46%)	[0–8.04]	
UC	Yes	4	2 (50%)	1.68	*1*
	No	11	4 (36%)	[0.089–32.5]	
CRT	Yes	3	1 (33%)	0.716	*1*
	No	12	5 (42%)	[0.010–17.6]	
Steroid	Yes	2	1 (50%)	1.55	*1*
	No	13	5 (38%)	[0.017–141]	
Anastomosis	Stapled	8	1 (13%)	13.6	*< 0.05*
	Hand-sewn	7	5 (71%)	[0.865–934]	
Distance of anastomosis from AV (cm)	< 4 4≤	6 9	4 (67%) 2 (22%)	0.167 [0.008–2.17]	*0.136*

*AL* anastomotic leakage; *POD* postoperative day; *BMI* body mass index; *ASA-PS* American Society of Anesthesiology physical status; *DM* diabetes mellitus; *UC* ulcerative colitis; *CRT* chemoradiotherapy; *AV* anal verge; *OR* odds ratio; *CI* confidential interval (*Fisher’s exact test)

We also examined hematological inflammatory findings on POD 7. In all patients, WBC and CRP were 7400 [5800–12,500] /μL and 1.75 [0.18–9.06] mg/dL, respectively. In patients with occult AL and without occult AL, WBC: 6900 [5800–12,500] /μL; CRP: 1.39 [0.18–9.06] mg/dL and WBC: 7500 [5800–11,200] /μL; CRP: 1.99 [0.60–8.32] mg/dL, respectively. Even in the occult AL group, neither WBC nor CRP were significantly elevated, nor were they helpful for diagnosis. In addition, all patients who were diagnosed with occult AL had no symptoms associated with AL during their postoperative course.

## Discussion

In patients who had undergone a low rectal resection with a DS for an anastomotic line less than 5 cm from the AV, the incidence of occult AL on POD 7 was 6 of 15 (40%), and compared with stapled anastomosis, hand-sewn anastomosis, was a statistically significant risk factor. With DRE and CE alone, it was difficult to detect occult AL, and five more cases with occult AL that could not be detected with CE alone were detected with CT following CE. In other words, CE alone had a 33% false-negative radiological result rate in this study.

Alves et al. reported that a 7.5% false-negative radiologic result rate leads to re-intervention in two-thirds of these patients in their randomized clinical trial of early stoma closure (EC) vs late (conventional timing) stoma closure [14]. In the trial, antegrade water-soluble CE through the distal limb of the DS was performed on POD 7, and the retrograde (transanal) approach was not used to avoid potential anastomotic injury; however, this risk remains unproven. Gouya et al. reported that CT antegrade colonography was more accurate than antegrade fluoroscopy for evaluation of both low anastomosis and surrounding space patency [17]. Danielsen et al. reported no false-negative radiologic results using CT with water-soluble CE [12].

Careful selection of the patients remains crucial to maintaining low overall postoperative morbidity, which is the aim of an early stoma reversal. In this regard, imaging plays a pivotal role because a false-negative radiologic result may lead to potentially dangerous EC, thereby increasing the risk of anastomotic septic complications. Considering the results of our study, CT following water-soluble CE is not a complicated method and could be useful in detecting occult AL for patients in whom early closure of the stoma is being considered.

In this study, other variables did not appear to be any correlation with the incidence of occult AL; however, these results were considered to be a result of the small sample size. Further confirmation is needed to assess the significance of these variables and to establish the optimal inclusion and exclusion criteria of EC.

All patients who were diagnosed with an occult AL had no symptoms associated with AL during their postoperative course. In addition, there were no significant elevations of WBC and CRP on POD 7 in this study; however, if EC had been performed for such patients, an occult AL may very well have developed into a symptomatic AL. To perform EC safely, both inclusion and exclusion criteria and methods to assess anastomosis should be standardized.

This study has several limitations. First, it is a single-center prospective observational study, with a very short study period and small overall sample size. For these reasons, we considered that it is statistically inappropriate to perform a multivariate analysis because such an analysis limits the accuracy of our outcome analysis. Second, as EC was not performed for this cohort, it is unproven whether radiological occult AL is correlated with any clinical symptoms after EC.

## Conclusions

The incidence of occult AL on POD 7 in patients with a DS was as high as 40%, and a hand-sewn anastomosis appeared to be a risk factor. When we considered the inclusion criteria of early stoma closure (EC), stapled anastomosis was preferred. As water-soluble CE alone may have high false-negative radiological result rates, CT following CE should be chosen as a standard method to assess anastomosis integrity before EC. If the safety of EC is ensured by appropriate indication and examination, EC may be adopted as the standard treatment in the future.

## Data Availability

The datasets used and/or analyzed during the current study are available from the corresponding author on reasonable request.

## Abbreviations

AL: Anastomotic leakage
ASA-PS: American Society of Anesthesiology physical status
AV: Anal verge
BMI: Body mass index
CE: Water-soluble contrast enema
CRP: C-reactive protein
CRT: Chemoradiotherapy
CT: Computed tomography
DM: Diabetes mellitus
DRE: Digital rectal examination
DS: Diverting stoma
EC: Early stoma closure
FAP: Familial adenomatous polyposis
IPAA: Total colectomy with ileal pouch-anal anastomosis
ISR: Intersphincteric resection
LAR: Low anterior resection
MIS: Minimally invasive surgery
POD: Postoperative day
TaTME: Transanal total mesorectal excision
UC: Ulcerative colitis
WBC: White blood cell
